# Prevalence of prehypertension and its relationship to risk factors for cardiovascular disease in Jamaica: Analysis from a cross-sectional survey

**DOI:** 10.1186/1471-2261-8-20

**Published:** 2008-08-28

**Authors:** Trevor S Ferguson, Novie OM Younger, Marshall K Tulloch-Reid, Marilyn B Lawrence Wright, Elizabeth M Ward, Deanna E Ashley, Rainford J Wilks

**Affiliations:** 1 Tropical Medicine Research Institute, University of the West Indies, Mona, Kingston 7, Jamaica; 2 Ministry of Health, Jamaica

## Abstract

**Background:**

Recent studies have documented an increased risk of cardiovascular disease (CVD) in persons with systolic blood pressures of 120–139 mmHg and/or diastolic blood pressures of 80–89 mmHg, classified as prehypertension in the Seventh Report of the Joint National Committee on Prevention, Detection, Evaluation, and Treatment of High Blood Pressure. In this paper we estimate the prevalence of prehypertension in Jamaica and evaluate the relationship between prehypertension and other risk factors for CVD.

**Methods:**

The study used data from participants in the Jamaica Lifestyle Survey conducted from 2000–2001. A sample of 2012 persons, 15–74 years old, completed an interviewer administered questionnaire and had anthropometric and blood pressure measurements performed by trained observers using standardized procedures. Fasting glucose and total cholesterol were measured using a capillary blood sample. Analysis yielded crude, and sex-specific prevalence estimates for prehypertension and other CVD risk factors. Odds ratios for associations of prehypertension with CVD risk factors were obtained using logistic regression.

**Results:**

The prevalence of prehypertension among Jamaicans was 30% (95% confidence interval [CI] 27%–33%). Prehypertension was more common in males, 35% (CI 31%–39%), than females, 25% (CI 22%–28%). Almost 46% of participants were overweight; 19.7% were obese; 14.6% had hypercholesterolemia; 7.2% had diabetes mellitus and 17.8% smoked cigarettes. With the exception of cigarette smoking and low physical activity, all the CVD risk factors had significantly higher prevalence in the prehypertensive and hypertensive groups (p for trend < 0.001) compared to the normotensive group. Odds of obesity, overweight, high cholesterol and increased waist circumference were significantly higher among younger prehypertensive participants (15–44 years-old) when compared to normotensive young participants, but not among those 45–74 years-old. Among men, being prehypertensive increased the odds of having >/=3 CVD risk factors versus no risk factors almost three-fold (odds ratio [OR] 2.8 [CI 1.1–7.2]) while among women the odds of >/=3 CVD risk factors was increased two-fold (OR 2.0 [CI 1.3–3.8])

**Conclusion:**

Prehypertension occurs in 30% of Jamaicans and is associated with increased prevalence of other CVD risk factors. Health-care providers should recognize the increased CVD risk of prehypertension and should seek to identify and treat modifiable risk factors in these persons.

## Background

The relationship between blood pressure and cardiovascular mortality is thought to be linear with no definite lower threshold that identifies potential danger or cessation of benefit [1,2]. Recent studies have documented an increase in the risk of cardiovascular disease and a high rate of progression to hypertension in persons with systolic blood pressures between 120 to 139 mmHg and/or diastolic blood pressures between 80 and 89 mmHg [2-4]. In addition, there have been reported associations between borderline hypertension (systolic 130–140 mm Hg or diastolic 85–89 mm Hg) and high normal blood pressure (systolic 130–139 mmHg or diastolic 85–89 mmHg) with several risk markers for cardiovascular events, such as increased carotid intima-media thickness, left ventricular hypertrophy and microalbuminuria [5-8]. Based on the current evidence, the Seventh Report of the Joint National Committee on Prevention, Detection, Evaluation, and Treatment of High Blood Pressure (JNC 7) [9] recommended a new classification for blood pressure in which normal blood pressure is defined as systolic blood pressure < 120 mmHg and diastolic blood pressure of < 80 mmHg, while persons with systolic blood pressure of 120 to139 mmHg and/or diastolic blood pressure of 80 to 89 mmHg are classified as having prehypertension. This new classification places a large number of persons previously considered as normal in this higher risk category and emphasizes the need for monitoring and possible intervention in persons with blood pressures between the range of normal and hypertensive.

Since the publication of JNC 7 a number of studies have reported associations between prehypertension and other risk factors for cardiovascular disease. These include obesity, high total cholesterol, diabetes mellitus [10], elevated C-reactive protein [11,12] and left ventricular hypertrophy [13]. One study reported an association between prehypertension and coronary atherosclerosis in persons undergoing coronary angiograms [14]. Little is known about the association between these risk factors and prehypertension in Jamaica.

To date, there are only a few reports of national prevalence estimates for prehypertension, with estimates ranging from 30% to 48.9% [15-19]. In most of theses studies, prehypertension was more common than hypertension. The prevalence of prehypertension in Jamaica is not known. However, hypertension is known to be common in Jamaica and other developing countries of the Caribbean region. Population-based studies have shown a prevalence of hypertension of approximately 20% in persons 15–74 years old in Jamaica [20].

The aim of this study was to estimate the prevalence of prehypertension from a population-based study – The Jamaica Lifestyle Survey [21]. In order to assess overall cardiovascular risk, we also examined the association between prehypertension and some known risk factors for cardiovascular disease, specifically diabetes mellitus, overweight or obesity, high-risk waist circumference and hypercholesterolaemia.

## Methods

The Jamaica Healthy Lifestyle Survey 2000–2001 studied 2012 persons between the ages of 15 and 74 years in order to estimate the prevalence of hypertension, diabetes and obesity in Jamaica. The details of the study design and conduct have been previously published [21]. Participants were asked to complete an interviewer-administered questionnaire and had anthropometric and blood pressure measurements performed by trained observers using standardized procedures. Fasting blood glucose and total cholesterol were measured using a capillary blood sample.

Blood pressure was measured from the right arm of the seated participant after five minutes rest and was recorded to the nearest 2 mmHg using 1st and 5th Korotkoff sounds. Three blood pressure measurements were taken and the mean of the last two measurements was used in the analysis. Height was measured with a portable stadiometer and recorded to the closest 0.1 cm. Waist circumference and hip circumference were measured with a non-stretchable tape measure to the nearest 0.1 cm. Weight was measured with an electronic digital scale to the nearest 0.1 kg. Measurements were conducted by trained personnel and all instruments were calibrated once weekly. Fasting blood glucose and total cholesterol were measured after a 10 hour overnight fast using a capillary blood sample analyzed with the Accutrend GCT Roche Diagnostics GmbH instrument.

The protocol for the study was reviewed and approved by Ethics Committees in the Faculty of Medical Sciences of the University of the West Indies, Mona and in the Ministry of Health, Jamaica.

### Definitions

Prehypertension was defined according to JNC 7 criteria as having either a systolic blood pressure of 120 to139 mmHg and/or diastolic blood pressure of 80 to 89 mmHg in persons who were not on treatment for hypertension. Hypertension was also defined according to JNC 7 criteria as having an untreated systolic blood pressure (BP) of greater than or equal to 140 mmHg or diastolic BP greater than or equal to 90 mmHg or being on medication for hypertension. Normal blood pressure was defined as having both a systolic BP of < 120 mmHg and a diastolic BP of < 80 mmHg in the absence of antihypertensive medication. Diabetes mellitus was defined as having a fasting glucose of greater than or equal to 6.1 mmol/L or being on treatment for diabetes [22]. Body mass index (BMI) was calculated as weight in kilograms divided by the square of the height in metres. Overweight was defined as a BMI greater than or equal to 25 kg/m^2^, while obesity was defined as BMI greater than or equal 30 kg/m^2 ^[23]. Increased (high-risk) waist circumference was defined as recommended by Lean et al. [24], as greater than 94 cm in men and greater than 80 cm in women. Increased waist to hip ratio was defined as greater than or equal to 0.95 for males and greater than or equal to 0.80 for females. Hypercholesterolaemia was defined as fasting total serum cholesterol of greater than or equal to 5.2 mmol/L (200 mg/dL). Cigarette smoking was defined as smoking one or more cigarettes per day.

Physical activity status defined using information obtained from the questionnaire. Low activity persons was defined as infrequent (< 3 times per week) involvement in energy expenditure at either work, traveling to work or leisure-time activities.

### Statistical Methods

Data analysis was carried out using Stata Version 9 [25]. Analysis was restricted to 1972 participants with blood pressure data. Individual weights were created to correct for discrepancies between the age-sex distribution of the sample and that of the Jamaican population of 15–74 year-olds [21]. Standard error estimates for population prevalence were adjusted to account for the multistage sampling design used in this study. The chi-squared statistic corrected for survey design determined evidence of statistically significant association between cardiovascular risk factors and blood pressure categories. The chi-squared test for trend identified statistically significant trends in prevalence of cardiovascular risk factors across ordinal blood pressure categories. Multivariate logistic regression analysis provided estimates of the odds of prehypertensive participants having given CVD risk factors as well as various clusters of risk factors, namely, central obesity (having a high-risk waist circumference), smoking cigarettes, having diabetes mellitus, having high cholesterol or being older than forty five years. Separate models were created for each cardiovascular risk factor. Each model estimated the relative increase in the odds of having the respective risk factor for prehypertensive persons compared with normotensive persons. Odds ratios from models with hypercholesterolaemia or diabetes as outcome variable were adjusted for age, sex and overweight status. Odds ratios from models with measures of obesity as the outcome variable were adjusted for age, sex, diabetes and hypercholesterolaemia. We report age-group specific estimates of these odds ratios from models that gave statistically significant evidence of an interaction of age with prehypertension.

To examine the clustering of risk factors for cardiovascular disease, we estimated the proportion of participants within blood pressure categories having one, two or three or more of the five aforementioned additional risk factors. We then used logistic regression models to calculate sex-specific, age adjusted odds ratios for having one, two, or three or more additional risk factors among prehypertensive participants compared to those with normal blood pressure.

## Results

Of the 1972 participants included in the analyses 661 (33.5%) were males and 1311 (66.5%) were females. The mean age was 36.3 ± 0.45 years with no sex difference. Selected characteristics of participants in the survey grouped according to blood pressure categories are shown in Table 1. With the exception of height, the mean values for the characteristics presented were significantly higher (p < 0.001) among the prehypertensive and hypertensive groups compared to those with normal blood pressure. There was also a statistically significant trend (p < 0.001) for higher means of all characteristics, except height, going from normal blood pressure to hypertension. Comparison of the mean values for the other variables of interest between those who were excluded because of a missing value and those included, revealed no differences except for age, where those with any missing values were younger than those with no missing values (37.7 vs. 41.3, p = 0.003).

**Table 1:** Means and standard error for selected characteristics of participants in the Jamaica Lifestyle Survey 2000–2001 within and across blood pressure categories.

		Total N = 1972	Normotensive* N = 875	Prehypertensive N = 555	Hypertensive N = 542	p-value for association**
		
Characteristics	N	Mean ± SE^#^	Mean ± SE	Mean ± SE	Mean ± SE	
Age (years)	1953	36.3 ± 0.45	30.3 ± 0.44	36.6 ± 0.73	50.2 ± 0.83	< 0.001
Weight (kg)	1939	71.5 ± 0.47	67.5 ± 0.55	74.0 ± 0.75	77.5 ± 1.01	< 0.001
Height (cm)	1936	167.1 ± 0.33	166.6 ± 0.39	168.6 ± 0.56	166.2 ± 0.54	0.002
Waist circumference (cm)	1944	82.3 ± 0.41	77.9 ± 0.48	84.1 ± 0.64	90.3 ± 0.86	< 0.001
Hip Circumference (cm)	1935	99.7 ± 0.46	97.1 ± 0.53	101.3 ± 0.62	103.8 ± 0.73	< 0.001
Waist to Hip Ratio	1932	0.82 ± 0.003	0.82 ± 0.003	0.83 ± 0.003	0.86 ± 0.005	< 0.001
Body mass index (kg/m^2^)	1930	25.7 ± 0.18	24.4 ± 0.22	26.1 ± 0.28	28.1 ± 0.38	< 0.001
Cholesterol level (mmol/L)	1848	4.5 ± 0.02	4.3 ± 0.2	4.5 ± 0.46	4.8 ± 0.06	< 0.001
Fasting Glucose (mmol/L)	1881	4.3 ± 0.05	4.0 ± 0.04	4.21 ± 0.07	5.0 ± 0.12	< 0.001

* Normotensive: blood pressure (BP) < 120/80 mmHg; Prehypertensive: BP = 120–139/80–89 mmHg and not on anti-hypertensive medication; Hypertensive: BP ≥ 140/90 or on anti-hypertensive medication

** Apart from height all characteristics showed a statistically significant increasing trend (P < 0.001) going from normotensive to hypertensive

#SE = Standard error

**Figure 1:**
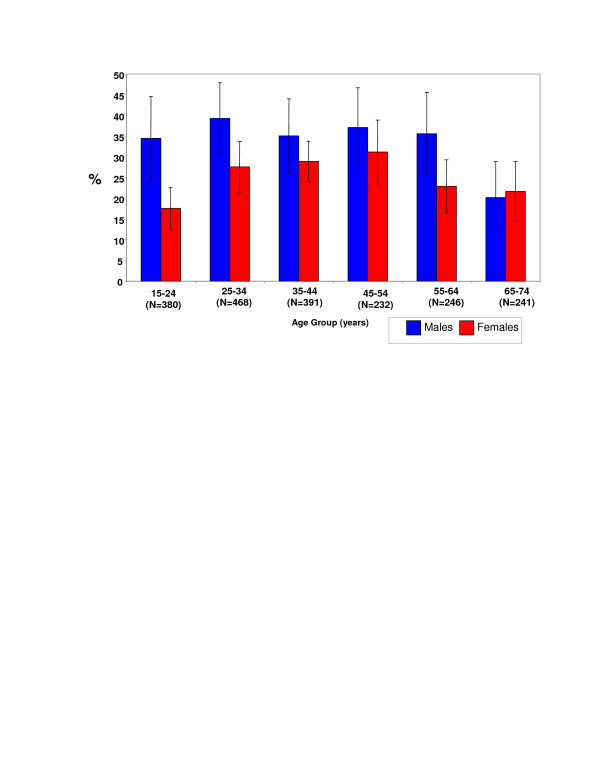
Age-group and sex specific prevalence of prehypertension (with 95% confidence intervals shown as error bars) in the Jamaica Lifestyle Survey 2000–2001.

The overall prevalence of prehypertension among Jamaicans 15–74 years old was found to be 30% (95% confidence interval [CI] 27%–33%). Prehypertension was more common in men than women, 35% (CI 31%–39%) and 25% (CI 22%–28%) respectively (p < 0.001 for male-female difference in proportion). Age and sex specific prevalence estimates are shown Figure 1. Prehypertension was more common in men than women at all ages except in the 65–74 year old age group. Among men, prehypertension prevalence ranged between 35–39% for the 15–64 year-old age groups then fell to 20% among those aged 65–74 years old (p for trend 0.04). Among women however, prehypertension prevalence was low at 18% among the youngest age group, and increased with age up to the 45–54 year-old age group, where the prevalence was 31%, after which there was a decline in the older age groups, 23% and 22% in the 55–64 year-old and 65–74 year-old age groups respectively (the test for trend was not significant).

Table 2 shows the crude and sex-specific prevalence of cardiovascular risk factors among participants in the study. Overall there was high prevalence of obesity in the study population. Almost 46% of participants were overweight (≥ 25 kg/m^2^); 19.7% were obese (≥ 30 kg/m^2^); 30.5% had increased waist/hip ratio while 36% had increased waist circumference. The prevalence low physical activity levels was estimated at 36.3%. Prevalence estimates were lower for other CVD risk factors: 14.6% for hypercholesterolemia, 7.2% for diabetes mellitus and 17.8% for cigarette smoking. There were marked sex differences in the prevalence of CVD risk factors except for diabetes mellitus. For measures of obesity, prevalence was two to nine times higher among women compared to men (p < 0.001 for all measures).

**Table 2:** Crude and sex-specific prevalence of cardiovascular disease risk factors in the Jamaica Lifestyle Survey 2000–2001.

Characteristics	Male (N = 661)	Female (N = 1311)	Total (N = 1972)
	
	% (95% CI)	% (95% CI)	% (95% CI)
Obesity** (BMI ≥ 30 kg/m^2^)	9.0 (6.8–11.3)	30.0 (26.8–33.2)	19.7 (17.4–22.0)
Overweight** (BMI ≥ 25 kg/m^2^)	30.2 (25.7–34.6)	60.7(57.4–64.0)	45.7 (42.6–48.9)
Increased Waist Circumference**	14.6 (11.6–17.6)	56.7 (53.2–60.2)	36.0 (33.0–39.0)
Increased Waist/Hip ratio**	6.1 (4.0–8.1)	54.4 (50.6–58.2)	30.5 (27.9–33.2)
Hypercholesterolaemia*	11.6 (8.8–14.5)	17.4 (15.1–19.8)	14.6 (12.7–16.5)
Diabetes Mellitus	6.3 (4.3–8.3)	8.0 (6.6–9.4)	7.2 (6.0–8.3)
^1^Cigarette Smoking**	28.3 (24.1–32.5)	7.3 (5.6–8.9)	17.6 (15.2–20.1)
Low Physical activity**	21.3 (17.3–25.4)	50.9 (46.8–54.9)	36.3 (33.0–39.7)

^1 ^Currently smokes cigarettes regardless of quantity per day.

* P < 0.01 (male – female difference in proportions)

** P < 0.001 (male – female difference in proportions)

Table 3 shows the sex-specific prevalence of CVD risk factors and risk factor clusters by blood pressure categories with assessment for trends moving from normal to high blood pressure. With the exception of cigarette smoking and low physical activity levels all the CVD risk factors studied had significantly higher prevalence in the prehypertensive and hypertensive groups compared to the normotensive group among both men and women. There were also statistically significant trends (p < 0.001) for higher prevalence going from normal to high blood pressure. Low physical activity was also associated with blood pressure among men with higher prevalence among those with prehypertension or hypertension. Among women however, the prevalence of low physical activity was high in all blood pressure categories. For cigarette smoking, persons with hypertension had lower prevalence of cigarette smoking compared to those with normal blood pressure or prehypertension among both men and women.

**Table 3:** Prevalence of individual cardiovascular disease risk factors and risk factor clusters by sex and blood pressure categories in the Jamaica Lifestyle Survey 2000–2001.

Individual Cardiovascular Disease Risk Factor								
	Male				Female			
	
	Normal BP % (95% CI)	Pre-HTN % (95% CI)	HTN % (95% CI)	P-value (trend)	Normal BP % (95% CI)	Pre-HTN % (95% CI)	HTN % (95% CI)	P-value (trend)
	
Obesity	4.8 (2.0–7.5)	8.6 (4.9–12.4)	19.5 (11.8–27.1)	< 0.001	19.7 (16.6–22.8)	39.0 (32.7–45.2)	45.4 (39.6–51.3)	< 0.001
Overweight	19.2 (14.4–24.0)	31.2 (24.9–37.5)	53.3 (43.3–63.4)	< 0.001	48.4 (44.1–52.8)	71.3 (65.8–76.9)	79.1 (75.2–83.3)	< 0.001
Increased Waist Circumference	6.3 (3.2–9.4)	13.7 (8.7–18.6)	34.6 (26.3–43.0)	< 0.001	42.4 (37.9–46.8)	66.9 (60.8–72.9)	81.1 (77.1–85.1)	< 0.001
Increased Waist/Hip ratio	2.6 (0.4–4.9)	4.7 (2.0–7.6)	16.2 (9.4–23.1)	< 0.001	42.1 (37.2–47.0)	60.4 (54.6–66.2)	78.1 (72.6–83.5)	< 0.001
Hypercholesterolaemia	5.6 (2.6–8.7)	13.7 (8.6–18.6)	21.4 (14.3–28.4)	< 0.001	11.4 (8.9–13.9)	21.5 (16.5–26.5)	27.7 (22.7–32.6)	< 0.001
Diabetes Mellitus	2.2 (0.6–3.8)	4.3 (1.9–6.6)	19.0 (12.1–25.9)	< 0.001	2.4 (1.3–3.5)	7.2 (4.8–9.5)	22.4 (17.5–27.2)	< 0.001
^1^Cigarette Smoking	31.7 (24.6–38.8)	28.8 (21.6–35.9)	19.8 (14.2–25.4)	0.004	8.0 (5.7–10.3)	6.8 (3.7–10.0)	5.9 (3.2–8.5)	0.033
Low Physical Activity	18.7 (13.1–24.3)	20.8 (14.1–27.4)	28.3 (20.4–36.2)	0.007	52.0 (47.2–56.9)	46.2 (39.8–52.7)	53.4 (46.2–60.6)	0.181

Risk Factor Clustering^2^								

	Male				Female			
	
	Normal BP % (95% CI)	Pre-HTN % (95% CI)	HTN % (95% CI)	P-value (trend)	Normal BP % (95% CI)	Pre-HTN % (95% CI)	HTN % (95% CI)	P-value (trend)
	
No Additional Risk Factor	54.1 (47.9–60.3)	45.6 (37.1–54.1)	19.6 (10.5–28.7)	< 0.001	48.4 (43.9–52.8)	22.6 (16.2–29.2)	6.7 (3.6–9.7)	< 0.001
1 Additional Risk Factor	33.1 (26.5–39.6)	28.0 (21.9–34.1)	29.1 (20.5–37.7)	0.225	33.6 (29.3–37.9)	40.9 (35.3–46.5)	26.1 (20.6–31.5)	< 0.001
2 Additional Risk Factors	11.0 (6.8–15.2)	19.6 (13.6–25.5)	30.4 (23.8–37.1)	< 0.001	13.8 (10.9–16.7)	25.8 (20.5–31.2)	32.9 (27.1–38.7)	< 0.001
≥3 Additional Risk Factors	1.8 (0.4–3.2)	6.8 (3.5–10.2)	20.9 (15.0–26.7)	< 0.001	4.2 (2.4–6.1)	10.6 (7.6–13.5)	34.3 (28.8–39.8)	< 0.001

^1 ^Currently smokes cigarettes regardless of quantity per day

^2 ^Risk factors include diabetes mellitus, hypercholesterolemia, central obesity, cigarette smoking and age > 45 years

The proportion of persons with clustering of CVD risk factors are also shown in Table 3. They are grouped as "no additional risk factor", "one additional risk factor", "two additional risk factors" and three or more additional risk factors"; and reported as sex-specific estimates by blood pressure category. Compared with normotensive men, those with prehypertension were more likely to have two additional risk factors (19.6% among prehypertensive vs. 11.0% among normotensive), or three or more additional risk factors (6.8% among prehypertensive vs. 1.8% among normotensive persons). Similarly, among women, a higher proportion of those with prehypertension had one, two and three or more risk factors compared to those with normal blood pressure. Almost 41% of prehypertensive women had one additional risk factor, 25.8% had two additional risk factors, and 10.6% had three or more risk factors, compared to 33.6%, 13.8% and 4.2% for the respective groups with normal blood pressure.

In order to estimate the relative risk for the presence of CVD risk factors among person with prehypertension compared to those with normal blood pressure, odds ratios were obtained using logistic regression models with adjustments for age and sex and other CVD risk factors as stated above. These odds ratios are shown in Table 4. There was significant effect modification with age and therefore age-group specific odds ratios were calculated. There was no significant association for the odds of diabetes mellitus among prehypertensive persons in any age group. Odds of obesity, overweight, high cholesterol and increased waist circumference tended to be significantly higher among young persons (15–44 year olds) with prehypertension compared to young persons with normal blood pressure. The odds ratio for increased waist/hip ratio was only significant among younger prehypertensive persons in the age group 15–24 years. In the older age groups (45 year olds and older), while the odds of the CVD risk factors were often higher among those with prehypertension the association was not significant except for increased waist/hip ratio in the 45–54 year-old age group.

**Table 4:** Age specific odds ratios (with 95% CI in brackets) from multivariate logistic regression models for cardiovascular disease risk factors among prehypertensive persons compared to normotensive persons in the Jamaica Lifestyle Survey.

	Age Group (years)					
	
Risk Factor	15–24	25–34	35–44	45–54	55–64	65 & older
Obese^+^	3.2(1.7–5.9)	1.7(1.01–2.8)	2.8(1.8–4.3)	1.8(0.8–3.9)	1.1(0.5–2.5)	0.9(0.2–3.5)
Overweight^+^	2.8(1.6–4.7)	2.2(1.3–3.6)	1.4(0.9–2.3)	1.3(0.6–2.8)	1.0(0.4–2.6)	1.1(0.3–3.5)
High cholesterol^++^	1.5(0.5–4.4)	2.0(1.1–3.7)	1.9(1.1–3.4)	2.1(0.8–5.2)	0.5(0.2–1.3)	0.8(0.3–1.9)
Diabetes^++^	0.7 (0.2–2.7)^+++^		0.7(0.2–2.7)	1.8(0.7–5.0)	2.4 (0.8–7.9)	4.1(0.8–19.8)
Increased Waist circumference^+^	3.1 (1.6–5.9)	2.0(1.1–3.3)	1.4(0.9–2.2)	1.6(0.8–3.3)	1.6(0.5–4.7)	1.6(0.4–5.7)
Increased^+ ^Waist/Hip ratio	1.9(1.1–3.2)	1.3(0.9–2.0)	1.03(0.6–1.8)	3.1(1.3–7.3)	1.01(0.5–2.2)	0.8(0.3–2.8)

^+ ^Odds ratios adjusted for sex, diabetes and high cholesterol status

^++ ^Odds ratios adjusted for sex and overweight status

^+++ ^Odds ratios for age range 15–34 since there were no diabetes cases in the 15–24 year-olds.

We also estimated the age-adjusted odds ratio for relative increase in odds for one, two, and three or more risk factors compared to having no risk factors for male and female prehypertensive participants compared to normotensive participants. Among men, being prehypertensive is associated with an almost three-fold (OR 2.8 [CI 1.1–7.2]) increase in the odds of having three or more risk factors, while there is no apparent increased odds associated with two (OR 1.4 [0.7–2.9]) or one (OR 0.8 [0.5–1.4]) CVD risk factors. Among women, being prehypertensive is associated with increased odds at all levels of other CVD risk factors, with an approximately two-fold increase in the odds at each level of risk. The odds ratio for having three or more risk factors was 2.0 (95%CI 1.3–3.0) while for two risk factors, the odds ratio was 2.3 (1.3–3.8) and for one risk factor, the odds ratio was 2.3 (1.4–4.0).

## Discussion

The results of this study indicate that prehypertension affects almost one third of the Jamaican population. In this study the prevalence was higher in males compared to females in all age categories up to age 64 years and was similar in men and women in the oldest age category. Prehypertension was associated with several other risk factors for cardiovascular disease with significantly higher prevalence of overweight, obesity, increased waist circumference, hypercholesterolemia and diabetes mellitus when compared to persons with normal blood pressure. In addition, persons with prehypertension were more likely to have multiple additional CVD risk factors when compared to those with normal blood pressure.

The findings in our study are generally consistent with other studies in the published literature. Prevalence estimates reported range from 31% in the United States, 31.6% in Korea, 34% in Taiwan, 40% in the Ashanti region of Ghana, to 47% in Liaoning Province in China and 48.9% among the military in Israel [15-19,26]. Except for the 48.9% in Israel and the 47% from Liaoning Province in China these figures are generally similar to our estimates for Jamaica. The prevalence of prehypertension is therefore high in both developed and developing countries and in eastern and western populations. This supports the view that the rates of chronic non-communicable diseases, in particular cardiovascular diseases, in developing countries are rapidly approaching the rates in developed countries and that their prevention requires urgent attention [27].

The association of prehypertension with multiple risk factors for cardiovascular disease has also been described in the American population where the odds of hypercholesterolemia, diabetes mellitus and overweight/obesity was found to be greater among persons with prehypertension compared to persons who were normotensive [10]. In Korea, persons with prehypertension were more likely to have the metabolic syndrome than persons who had normal blood pressure [15]. In addition the study from Israel found that both men and women with prehypertension were more likely to have hyperglycemia, dyslipidaemia, obesity, the metabolic syndrome and a > 15% ten year risk of CVD when compared to persons with normal blood pressure [16]. This clustering of cardiovascular disease risk factors among persons with prehypertension suggest that persons found to have prehypertension should be screened for other cardiovascular disease risk factors, regardless of age.

The higher prevalence of prehypertension among men (particularly younger men) compared to women is noteworthy. This finding of higher blood pressures among younger men compared to women has been previously reported in the Caribbean [28]. Overall, the high prevalence of prehypertension in the younger age groups suggest that although the absolute risk of CVD attributable to prehypertension may be low, the population attributable risk may be relatively high and therefore will have important public health implications.

Based on data from The Statistical Institute of Jamaica [29] the thirty percent prevalence of prehypertension represents approximately seven hundred and eighty thousand persons in Jamaica in this age range. Prior to the publication of JNC 7 these persons would have been considered to have "normal" blood pressure and may not have been recognized as potential candidates for cardiovascular intervention or risk reduction. We now know however, that persons with prehypertension are at increased risk of cardiovascular events including progression to hypertension. The evidence that these persons are at risk is now quite convincing and some studies have begun to evaluate the role of pharmacological intervention [30]. The need for intervention, whether lifestyle or pharmacological is supported by the findings of Russell et al. who estimated that among adults 25–74 years old in the United States, prehypertension alone accounted for 3.4% of hospitalizations, 6.5% of nursing home admissions and 9.1% of deaths [31]. These data suggest that prehypertension should probably be included in the various instruments for estimating cardiovascular risk among persons in the lower risk groups.

The public health implication of this increased burden of at-risk people in our population is worthy of serious evaluation. It has been estimated that a 5 mmHg reduction in systolic blood pressure in the population will produce a 14% reduction in the risk of stroke and a 9% reduction in the risk of coronary heart disease [9]. If we apply a population approach to disease prevention we could therefore expect that a small reduction in mean population blood pressure will result in relatively large reductions in overall CVD risk. This can be achieved through either lifestyle or pharmacological intervention. It is unlikely that developing countries can afford pharmacological intervention [30] and lifestyle intervention [9,32,33] may be difficult to implement and maintain. Any intervention strategy will therefore require a multi-level, multi-sectorial approach [34-36].

The cross-sectional design of our study prohibits any causal inference from the identified associations as a time-sequence relationship cannot be determined. The study was population based and the sample was selected to be representative of the general Jamaican population. Where particular segments were not adequately represented weights were added to correct this prior to statistical analysis. One other possible limitation of this study is that there were missing data for some variables used in the analysis. The proportion of missing variables was however relatively small ranging from 3–8%. In addition there were no significant differences in the mean values of demographic and available CVD characteristics, except for age, for those with any missing data compared to those with no missing data. We are therefore satisfied that the estimates presented reflect the situation in the general Jamaican population. Given the similarities between the Jamaican population and other countries of the English speaking Caribbean, it is likely that these findings are applicable to the other countries of the Caribbean region.

## Conclusion

Prehypertension is highly prevalent in Jamaica and clusters with other cardiovascular disease risk factors. Health care providers and health planners should be made aware of the large numbers of persons at increased risk for cardiovascular disease and steps should be taken to identify and treat modifiable risk factors in such persons. At the very least a proper diet and regular exercise should be recommended. Further studies are needed to determine the rate of cardiovascular events in the prehypertensive population and the impact of various interventions on the rates of these cardiovascular events.

## Competing interests

The authors declare that they have no competing interests.

## Authors' contributions

TSF wrote the initial and revised manuscript and assisted with statistical analysis and interpretation of data; NOMY contributed to the design and conduct of original survey, performed statistical analyses and critically reviewed manuscript; MKT-R critically reviewed manuscript and assisted with statistical analyses and interpretation of data; MBLW critically reviewed manuscript and assisted with interpretation of data; EMW contributed to the design and conduct of original survey and critically reviewed manuscript; DEA contributed to the design and conduct of original survey and critically reviewed manuscript; RJW conceived, designed and managed the original survey; contributed to the interpretation of data and critically reviewed the manuscript and is the guarantor for the manuscript.

## Pre-publication history

The pre-publication history for this paper can be accessed here:


